# Evaluating fertility preservation interventions for alignment with ASCO Guidelines for reproductive aged women undergoing cancer treatment: a systematic review

**DOI:** 10.1007/s00520-023-08133-3

**Published:** 2023-11-11

**Authors:** Sarita Pathak, Susan T. Vadaparampil, Megan E. Sutter, Whitney S. Rice, Colleen M. McBride

**Affiliations:** 1 Department of Behavioral, Social, and Health Education Sciences, Emory University Rollins School of Public Health, Atlanta, GA USA; 2 Department of Health Outcomes and Behavior, Moffitt Cancer Center, Tampa, Florida USA; 3 Center for Reproductive Health Research in the Southeast, Emory University Rollins School of Public Health, Atlanta, GA USA

**Keywords:** Oncofertility, Fertility preservation, ASCO Guidelines, Reproductive-aged women, Interventions, Multi-level framework

## Abstract

**Purpose:**

While cancer treatment advancements have increased the number of reproductive-aged women survivors, they can harm reproductive function. Despite national guidelines, oncofertility service uptake remains low. This review explores interventions for fertility preservation alignment with American Society of Clinical Oncology (ASCO) guidelines and consideration of a multilevel framework.

**Methods:**

We systematically reviewed literature from 2006 to 2022 across four databases. Identified interventions were assessed and scored for quality based on CONSORT and TREND statement checklists. Results were synthesized to assess for intervention alignment with ASCO guidelines and four multilevel intervention framework characteristics: targeted levels of influence, conceptual clarity, methodologic pragmatism, and sustainability.

**Results:**

Of 407 articles identified, this review includes nine unique interventions. The average quality score was 7.7 out of 11. No intervention was guided by theory. Per ASCO guidelines, most (*n*=8) interventions included provider-led discussions of treatment-impaired fertility. Fewer noted discussions on fertility preservation approaches (*n*=5) and specified discussion timing (*n*=4). Most (*n*=8) referred patients to reproductive specialists, and few (*n*=2) included psychosocial service referrals. Most (*n*=8) were multilevel, with five targeting three levels of influence. Despite targeting multiple levels, all analyses were conducted at the individual level. Intervention strategies included: educational components (*n*=5), decision aids (*n*=2), and nurse navigators (*n*=2). Five interventions considered stakeholders’ views. All interventions were implemented in real-world contexts, and only three discussed sustainability.

**Conclusions:**

This review identifies key gaps in ASCO guideline-concordant fertility preservation that could be filled by updating and adhering to standardized clinical practice guidelines and considering multilevel implementation frameworks elements.

**Supplementary Information:**

The online version contains supplementary material available at 10.1007/s00520-023-08133-3.

## Background

Approximately one million women are diagnosed with cancer each year in the USA. As of 2020, 10% of these cancer cases occurred among women under the age of 40, with more than 48,000 new cancer diagnoses in adolescent and young adult (AYA) women ages 15 to 39 [1]. Substantial advances in early detection (e.g., screening uptake, genetic testing) and treatment (e.g., radiation therapy) for cancer has dramatically increased survivorship with greater than 85% of women diagnosed under age 45 surpassing 5-year survival rates [1–4].

To achieve these impressive outcomes, cancer treatments typically comprise extensive chemotherapy, radiotherapy, hormone therapies, and/or surgical procedures. While life-saving, these therapies can damage reproductive function [5–7]. For women, commonly used cancer treatments accelerate follicle and oocyte depletion, leading to impaired reproductive endocrine function and infertility. It is estimated that over 100 million women worldwide are at risk of cancer treatment-related ovarian impairments and may seek fertility preservation by 2025 [8].

Moreover, loss of endocrine support for hormonally responsive tissues can trigger a cascade of long-term medical problems in addition to infertility [9]. For example, existential psychosocial concerns and poor quality of life is prevalent and persistent in cancer patients and survivors [10, 11]. A recent review found factors such as early-onset menopause and unfulfilled desire for biological children are associated with poorer mental health outcomes in survivorship [12]. Some patients even view the loss of the ability to have biological children as more distressing than the cancer diagnosis itself [9, 12, 13]. One study found that approximately 50% of young cancer patients ages 18-45 who wished to have children in the future required some psychological support with regard to fertility and future parenthood after a cancer diagnosis [12, 14].

In 2006, the American Society of Clinical Oncology (ASCO) endorsed evidence-based clinical practice guidelines on fertility preservation for healthcare providers, publishing subsequent updates in 2013 and 2018. Aligning with other national and international recommendations, ASCO guidelines recommend that all patients undergoing potential gonadotoxic treatments should receive information and counseling about the impact of their disease or treatment on future fertility and fertility preservation options as part of their initial comprehensive care plan. Specifically, the guidelines encourage (1) provider discussion of potential impairment to fertility, (2) provider discussion of fertility preservation approaches, (3) all discussions occurring as early as possible prior to beginning treatment, (4) patient referral to reproductive specialists for fertility preservation, (5) documentation of fertility-related discussions, and (6) patient referral to psychosocial services for additional support [13].

To further facilitate care of patients at risk of exposure to gonadotoxic agents, fertility preservation options are outlined as part of these guidelines. Standard fertility preservation options vary based on several factors including patient’s biological sex, age and cancer type [5]. For example, guidelines recommend sperm cryopreservation or testicular tissue freezing for adult men. All of these procedures can be conducted quickly prior to the beginning of treatment. The only fertility preservation options recommended by the most recent ASCO guidelines for prepubertal children include ovarian or testicular cryopreservation, both of which are currently investigational procedures. Finally, fertility preservation options for reproductive age women include oocyte cryopreservation, embryo cryopreservation, ovarian shielding and transposition, ovarian tissue transplantation, etc. For most of these procedures, ovarian stimulation requires an average of 12 days to harvest oocytes prior to initiation of cancer treatments [3, 13].

Accordingly, many fertility preservation interventions have been implemented in recent years to increase the uptake of fertility preservation. However, approximately 30–50% of patients do not receive adequate information regarding infertility risks and preservation options prior to beginning cancer treatments [1]. A recent study reported that only 4 to 41% of females undergo preservation procedures [14]. Further, health care providers’ awareness of the need to talk about risks to fertility, fertility preservation options, and referral to reproductive specialists in a guideline concordant manner continue to remain low. Research to explore provider awareness of and adherence to ASCO guidelines has been limited and is somewhat dated. One study published in 2009 showed that of the approximately 60% of oncologists who reported awareness of ASCO guidelines, less than 25% reported following the guidelines on a regular basis, distributing educational materials, or referring patients for further discussions on fertility preservation [15, 16]. Another study published in 2011 found that of the oncologists who provided care to pediatric patients reported, only 44% were familiar with ASCO recommendations for fertility preservation [1, 17].

As envisioned by ASCO, guideline-concordant fertility preservation care is inherently multifaceted and influenced by a complex and interconnected set of barriers and facilitators operating at individual, interpersonal and organizational level. For example, in addition to the patient, various family members (e.g., parents and/or intimate partners) may need to be involved with decision-making. Further, a variety of health care providers (e.g., primary care, oncology, reproductive endocrinology, nursing, surgery, mental health) are also involved to address patients’ needs [7]. At the organizational level, variations in access, availability, and insurance coverage for reproductive healthcare additionally contributes to health-system barriers to fertility preservation. Additionally, social barriers such as structural racism mean that patients of color are less likely to be seen in cancer specialty settings, have access to and undergo fertility preservation when compared to White patients [14].

To evaluate the range of evidence-based interventions being used to implement ASCO guideline- concordant fertility preservation care, we conducted a systematic review assessing: (1) strategies and outcomes aligned with ASCO 2018 practice guidelines, (2) consideration of multilevel influences on service provision, and (3) sustainability of the evaluated interventions for healthcare system integration. To this end, we reviewed the literature published from 2006 to 2022 focusing on reproductive age women.

## Methods

### Defining intervention

In this review, we define interventions to include those testing strategies to increase consideration of and access to fertility preservation. Further, only articles in which interventions were evaluated relative to a comparison group are included.

### Data sources and searches

In conducting the review, we followed the Preferred Reporting Items for Systematic Reviews and Meta-Analysis (PRSMA) guidelines [18]. We completed systematic literature searches in four electronic databases (PubMed, Embase, Web of Science, Scopus) for intervention studies published between January 2006 (to align with publication of first ASCO guidelines related to fertility preservation [13] to January 2022. Keywords included: cancer, oncology, fertility preservation, oncofertility, intervention, healthcare utilization, and healthcare delivery. Full search terms can be seen in Appendix 1. We also hand searched any protocol papers to identify new articles subsequently published that were not captured in the initial search. We used Covidence to organize and manage the review database.

### Intervention study selection

The intervention study inclusion criteria were: English language, published between 2006 and 2022, that described an intervention including women of reproductive age (ages 13–49) with a cancer diagnosis and/or the providers that care for them. Recent data show the average age for the onset of menarche is at 12.4 years old. Thus, we selected 13 years old as the lower age limit to identify women of “reproductive age” [19]. Additionally, given that fertility preservation options vary by sex and age, we focused on reproductive age women. Excluded intervention studies (henceforth, “interventions”) were those that only included males or pediatric patients, were not accessible in full text, conference abstracts, study protocols, and reviews.

Using these criteria, we conducted initial title searches to identify articles for initial abstract review. Keywords were clarified to ensure that all relevant articles appeared in the search. Next, one author (SP) reviewed all titles and abstracts to identify articles for full-text review. Finally, we completed full-text reviews to determine the interventions to be included. Two authors (SP, CMM) completed data abstraction on all full-text review articles. All discrepancies were resolved through discussion to reach consensus.

### Quality assessment and data extraction

We applied the CONSORT [20] and TREND [21] statement checklists to assess intervention quality and the extent to which articles aligned with quality and reporting guidelines. If articles mentioned any of the concepts included on our checklist, we considered the criteria fulfilled. All articles were then scored for fulfilled criteria on a scale ranging from 0 (lowest quality)–11 (highest quality). Abstracted data were organized based on assigned quality score from highest to lowest.

We used the population, intervention, comparator, outcomes, timeframe, and study design (PICOTS) framework [22] to select which intervention characteristics were extracted. General intervention characteristics included: intervention study design, sample targeted, unit of analysis, intervention time period, in what country or geographic region the intervention took place, intervention setting (e.g., oncology clinic, fertility clinic), sample size, and general sample characteristics. In this review, healthcare providers included oncologists, reproductive specialists, surgeons, and nurses.

For each intervention, we determined the alignment of intervention targets and relevant outcomes with the ASCO guidelines for fertility preservation prior to cancer treatment. A “yes” was assigned for the following guideline elements were targeted as an intervention strategy or assessed as an outcome: (1) provider-led discussion of potential impairment to fertility, (2) provider-led discussion of fertility preservation approaches, (3) timing of discussions occurring prior to beginning treatment, (4) patient was referred to reproductive specialist for fertility preservation, (5) fertility-related discussion was documented, and (6) patient was referred to psychosocial services for additional support [13].

We coded interventions for their alignment with a parsimony-based operational framework based on complexity theory and pragmatic trials [23]. The framework considers four characteristics: socioecological levels of influence targeted, conceptual clarity, methodologic pragmatism, and sustainability. We noted the inter-level mechanisms implied at each level of influence that were targeted by the fertility preservation intervention and whether this was based on stakeholder input. For methodological pragmatism, we evaluated whether interventions took place in real world contexts and what background factors were considered in the evaluation of the intervention to enhance the generalizability of effectiveness findings. We also examined whether the interventions had any discussion or consideration of sustainability factors. Lastly, we noted whether the intervention outcome assessment showed significant benefit of the intervention for the proscribed outcomes.

### Data synthesis and analysis

Extracted data were organized by quality assessments for: general intervention characteristics, alignment of intervention strategies and outcomes with ASCO guidelines and consideration of multiple levels of influence. Within each category, we rated intervention quality from 0 to 11.

## Results

**Fig. 1:**
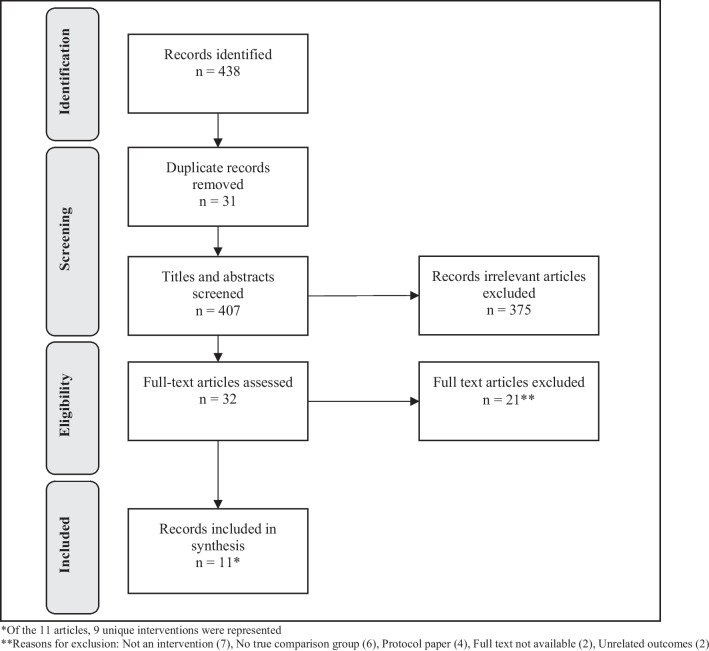
PRISMA statement. *Of the 11 articles, 9 unique interventions were represented. **Reasons for exclusion: Not an intervention (7), No true comparison group (6), Protocol paper (4), Full text not available (2), Unrelated outcomes (2)

We identified 438 articles from four electronic databases, after removing duplicates, there were 407 unique articles, 32 were eligible for full-text review after title and abstract screening, and 11 articles fit inclusion criteria for final data abstraction (see Fig. 1). All articles were published between 2012 to 2021. Among the 11 articles included, we identified nine unique interventions. The remainder of the results will be reported by intervention.

### Quality assessment

Among the nine included interventions, the highest quality score assigned was 9 (*n*=4 interventions) and the lowest was 5 (*n*=1). All interventions reported inclusion criteria, described group assignment, reported baseline and group comparisons, and reported measured outcomes. Most interventions detailed sampling methods (*n*=7) [24–31], provided sufficient detail for replicability based on CONSORT/TREND requirements (*n*=7) [24–31], and reported on subgroup analyses (*n*=5) [25, 26, 28–30, 32, 33]. Few interventions explained power considerations (*n*=4) [25–27, 29, 32, 33] and reasons for missingness in addition to how missing values were handled (*n*=2) [27, 28]. No intervention fulfilled all 11 criteria, and none reported a specific theory that guided the intervention. See Table 1.

**Table 1:** Quality assessment of interventions

	Introduction	Intervention Design				Statistical Methods					Results	Total**
Article	Use of Theory	Sampling Method	Power	Inclusion	Group Assignment	Replicability	Baseline comparisons	Group comparisons	Subgroup analyses	Missingness	Outcomes reported	
Ehrbar V, et al (2019) [25]		X	X	X	X	X	X	X	X		X	9
Ehrbar V, et al (2021) [26]												
Kelvin JF, et al (2016) [28]		X		X	X	X	X	X	X	X	X	9
Partridge AH, et al (2019) [29]		X	X	X	X	X	X	X	X		X	9
Peate M, et al (2012) [27]		X	X	X	X	X	X	X		X	X	9
Bradford NK, et al (2018) [30]		X		X	X	X	X	X	X		X	8
Srikanthan A, et al (2016) [31]		X		X	X	X	X	X			X	7
Vu J, et al (2017) [24]		X		X	X	X	X	X			X	7
Balcerek M, et al (2020) [33]			X	X			X	X	X		X	6
Borgmann-Staudt A, et al (2019) [32]												
Warner E, et al (2020) [34]				X	X		X	X			X	5

*Articles are shown in order of quality assessment

**Total out of 11 categories

### Intervention study characteristics

Descriptions of the interventions are detailed in Table 2. Most interventions utilized a quasi-experimental design (*n*=7) [24, 27, 28, 30–34] without randomization to a comparison group . Two interventions were randomized control trials [25, 26, 29]. The majority of the interventions focused on educational components to increase patient and/or provider knowledge regarding fertility preservation options and to assist in discussions and the decision-making process. This primarily targeted the individual and interpersonal levels of influence. Two interventions utilized decision aids to assist patients further in their ability to have discussions around fertility preservation options and to prevent decisional regret in the future [25–27]. Two interventions also leveraged the support of a nurse/patient navigator [24, 31]. Three took place in the USA [24, 28, 29]. Other settings included: Austria, Australia, Canada, the Czech Republic, Germany, Poland, and Switzerland. The majority of interventions were implemented in specialty cancer centers or healthcare practices, with one in regional fertility centers [25, 26]. Two interventions included patients as young as 12 [24, 32, 33]. Three interventions included males in the target sample [28, 30, 32, 33], one included patients’ parents [32, 33], and one specifically targeted breast surgeons [34]. Interventions typically did not focus on a particular cancer type, but when they did, breast cancer was most commonly included (*n*=6) [24–27, 29, 31, 34]. Five interventions did not report race/ethnicity of the sample [27, 30–34]; among those that reported the majority of participants were White. Most participants were in a relationship, had a college education, and were childless or had one child.

**Table 2:** General intervention characteristics

Article	Study Design	Intervention Description	Comparison Group	Sample Targeted	Unit of Analysis	Time Period	Country	Study Setting	Sample Size	Sample Characteristics	Type of Cancer
Ehrbar V, et al (2019) [25]	Randomized control trial	Patients were referred to reproductive specialists for counselling on fertility preservation and received an online decision aid immediately after	Counselling only control	Women ages 18-40 with a recent cancer diagnosis that potentially endangers fertility	Patient	2016-2017	Switzerland and Germany	Eight fertility centers	T1: 51 T2: 45 T3: 37	German women; Mean age 29.3; majority nulliparous and in a relationship	Variety, majority breast cancer
Ehrbar V, et al (2021) [26]											
Kelvin JF, et al (2016) [28]	Quasi experimental	The intervention included resources for patients (written educational materials and access to financial assistance), resources for clinicians (informational website, referral network, and a defined referral process), ongoing clinician education, fertility clinical nurse specialist consultations for patients, decision making assistance, facilitation of referrals, and clinical research and quality improvement	Historical control	Patients ages 18-45 at start of cancer treatment	Clinic Patient	Cohort 1: 2007-2008 Cohort 2: 2010-2012	United States (New York)	Memorial Sloan Kettering Cancer Center	Cohort 1: 150 males, 271 females Cohort 2: 120 males, 320 females	Majority White, college educated, Males and Females, average ages, 34.6, 37.9, respectively: in a relationship with children	Predominantly Testicular cancer, Lymphoma, Breast cancers
Partridge AH, et al (2019) [29]	2-arm clustered Randomized Control Trial	Women received a “Young women intervention” booklet, check-list for physician discussions, website access including videos and downloadable PDFs. Providers received parallel materials and email access to medical experts	Contact time comparison: Compared to a physical activity intervention	Women ages 18-45 at time of diagnosis	Patient	2012-2013	United States	Academic institutions (n=14) and Community sites (n=40)	Intervention arm: 245 Attention control arm: 222	Majority White; College educated; Age range 22-45;	Breast cancer
Peate M, et al (2012) [27]	Quasi experimental	Intervention patients received a decision aid containing information on breast cancer care and fertility with discussions on different fertility preservation options prior to any oncology or fertility specialist appointments	Historical control	Premenopausal women ages 18-40 interested in having children	Patient	2006-2009	Australia	Oncology Clinics	Intervention arm: 36 Historical control arm: 60	Race not noted, majority some college; mean age 33; 69% childless; 75% in committed relationship	Breast cancer
Bradford NK, et al (2018) [30]	Quasi experimental	The intervention comprised referral pathways; targeted education sessions for health professionals; and patients were provided with resource packets	Historical control	Cancer patients ages 14-25	Patient	2012-2015	Queensland, Australia	Five tertiary cancer centers	Pre-intervention Cohort: 260 Post-Intervention Cohort: 216	Race not noted; Over half ages 20-25; 59% male	Leukemia or lymphoma predominant cancer types
Srikanthan A, et al (2016) [31]	Quasi experimental	PYNK breast cancer program for young women includes a dedicated nurse navigator who recruits eligible patients as soon as a referral is received, is responsible for standardizing and coordinating care, facilitating decision-making, and providing education and personalized support throughout treatment and follow-up care	Historical control	Adult women ages 40 and younger at time of diagnosis and received chemotherapy	Patient	2011-2013	Canada	Cancer center clinics	81	Race not noted; Age range 21-40; college educated majority 0-1 child	Majority stage 2 & 3 Breast cancer
Vu J, et al (2017) [24]	Quasi experimental	Multifaceted program including outreach and education for providers, patient navigator support, a 24-hour fertility preservation hotline available to both patients and providers, online educational materials, and updated EMR requirements for provider-led discussions	Historical control	Women patients ages 45 and younger	Patient	2004 -2012 Intervention implemented 2007	US (Chicago)	Northwestern Comprehensive Breast Center	Pre-intervention cohort: 278 Post-intervention cohort: 515	Majority White; Median age for both cohorts 41, majority were married/partnered, about 30% in both groups did not have any children	Breast cancer
Balcerek M, et al (2020) [33]	Quasi experimental	PanCareLIFE program in which physicians shared a flyer with adolescents on fertility impairment with individual treatment-related fertility risk estimate (low, elevated or high), followed by brief discussion; Parents received a brochure on fertility impairment	Historical control	Adolescents with newly diagnosed cancers ages 12-19 undergoing chemotherapy/radiotherapy treatments	Patient Parent Provider	2014-2017	Austria, Czech, Germany, Poland	Pediatric oncology departments and clinics	101	Race not noted 60% male; 58% ages 13-15	Variety
Borgmann-Staudt A, et al (2019) [32]											
Warner E, et al (2020) [34]	Quasi experimental	Knowledge-translation intervention including a toolbox for breast surgeons with a 90-minute video seminar, informational one-pager, knowledge updates, management checklists, and a new patient survey	Usual care control	Breast cancer surgeons seeing women at cancer diagnosis and age 40 or younger who have not completed families	Provider	2014-2015	Canada	Health care practices that serve women diagnosed with breast cancer	Intervention arm: 27 Comparison arm: 56	Race not noted; Average age 50, average; Mean 17 years surgical experience	Breast cancer

*Articles are shown in order of quality assessment

### Alignment of intervention strategies and outcomes with ASCO guidelines

We assessed the extent to which the intervention strategies and relevant outcomes aligned with ASCO guidelines’ six expectations of fertility preservation service provision (See Table 3).

**Table 3:** Alignment of ASCO guideline components with interventions strategies and outcomes*

Article	Components of Practice Guidelines (Oktay 2018)					
	Discussions regarding fertility and potential impairment to fertility	Discussions regarding fertility preservation approaches	Address as early as possible before treatment starts	Discussion documentation	Referral to reproductive specialists (REI)	Referral to psychosocial services
Ehrbar V, et al (2019) [25]		X ^I^	X ^I^		X ^I^	
Ehrbar V, et al (2021) [26]						
Kelvin JF, et al (2016) [28]	X ^I,O^	X ^I,O^	X ^I^		X ^I,O^	
Partridge AH, et al (2019) [29]	X ^I,O^	X ^I,O^	X ^I^	X ^I,O^	X ^I,O^	X ^I,O^
Peate M, et al (2012) [27]	X ^I,O^	X ^I,O^			X ^I,O^	
Bradford NK, et al (2018) [30]	X ^I,O^			X ^I,O^	X ^I,O^	
Srikanthan A, et al (2016) [31]	X ^I,O^		X ^I^	X ^I,O^	X ^I,O^	X ^I^
Vu J, et al (2017) [24]	X ^I,O^	X ^I,O^		X ^I^	X ^I,O^	
Balcerek M, et al (2020) [33]	X ^I,O^					
Borgmann-Staudt A, et al (2019) [32]						
Warner E, et al (2020) [34]	X ^I,O^				X ^I^	

* ^I^ denotes intervention strategy; ^O^ denotes relevant outcomes that were measured

*Guideline Element 1: Health care providers should initiate discussion of the potential impairment to fertility.* The majority of the interventions (*n*=8) included strategies to assist providers in initiating discussion of potential treatment effects on fertility and measured the occurrence of these discussions as an outcome [24, 27–34]. To facilitate these discussions, one intervention included a 24-h fertility preservation hotline available to providers [24]. Additionally, the electronic medical record (EMR) was modified to prompt oncologists initiate discussions with their patients and required them to document discussion before the patient chart could be closed [24].

*Guideline Element 2: Providers discuss fertility preservation with patients.* Over half (*n*=5) of the interventions noted discussions regarding fertility preservation approaches [24–29], with one targeting discussion as an intervention strategy without measuring it as an outcome [25, 26]. The two most common intervention strategies to guide these discussions was providing patients with educational materials on fertility preservation [25–27] and providing patients with decision aids to review the options [24, 28, 29].

*Guideline Element 3: Provide fertility preservation services as early as possible prior to the patient beginning treatment.* Only four interventions specified that discussions occurred prior to treatment, and all four mentioned this as part of an intervention strategy but not as an outcome [25, 26, 28, 29, 31]. None of the interventions actually measured when the treatment discussions were occurring or provided description of what was done to promote early discussions.

*Guideline Element 4: Discussions of fertility effects and preservation be documented. *Four interventions noted that fertility-related discussions were documented [24, 29–31], however one did not measure documentation as an outcome [24]. In one intervention, a nurse navigator was utilized to screen referrals to the cancer center, expedite tests and consultations, and provide ongoing support to patients. The navigator documented all activities and services in the nursing section of the EMR [31].

*Guideline Element 5: Referral to fertility preservation services.* In the majority of interventions, providers referred patients to reproductive specialists either to discuss fertility preservation options or to receive fertility preservation treatment (*n*=8) [24–31, 34].

 Guideline Element 6: Refer patients for psychosocial services. This was noted in only two interventions [29, 31] with only one including referral as a measurable outcome [29]. As secondary outcomes, Partridge AH, et al (2019) examined attention to psychosocial concerns including noting emotional health, distress, referral for psychosocial support, etc. [29].

### Intervention framework alignment: levels of influence, inter-level mechanisms, stakeholder inclusion, methodological pragmatism, intervention effectiveness, sustainability

All interventions except one [27] were multilevel, with three targeting two levels (individual and interpersonal) [25, 29, 32, 33] and five targeting three levels (individual, interpersonal, and organizational/system/regional) [24, 28, 30, 31, 34]. Despite targeting multiple levels, all intervention effectiveness testing was conducted at the individual level. Primary outcomes included decisional conflict [25–27], fertility-related discussion and documentation of discussions [24, 29–31, 34], patient knowledge [32, 33], and patient satisfaction with intervention materials [28]. Eight interventions reported a significant effect for the primary outcome of intervention [24–28, 30–34].

When considering inter-level mechanisms for conceptual clarity, approximately half of the interventions included stakeholders’ views on the assumed process chains being targeted (*n*=4) [27, 28, 30, 31, 34]. Examples of stakeholder inclusion were utilizing multidisciplinary expertise to develop and pilot survey instruments, conducting focus groups with the patient population prior to the intervention, pilot testing decision aids, conducting a provider needs assessment to develop education tools and intervention materials, and establishing an interdisciplinary steering committee to guide intervention development and implementation.

When considering methodologic pragmatism, all interventions were implemented in real world contexts as they took place in cancer centers, healthcare practices, and fertility clinics. Across the targeted levels of influence, seven interventions considered the sociodemographic characteristics of the patients as background factors [24–29, 31, 34], one intervention considered literacy level differences by region [30], with only one intervention making no mention of background factors [32, 33].

Only three of the 11 interventions discussed sustainability [25–28]. Examples of sustainability included easily updating and/or transitioning to online decision aid materials and considering strategies to increase physician use of resources. Two interventions explored ideas for how they could make the intervention more sustainable in the future [24, 31]. All components of the multilevel framework are outlined in Table 4.

**Table 4:** Evaluating interventions utilizing a multilevel framework

Article	Intervention Characteristics			Conceptual Clarity			Methodologic Pragmatism	Sustainability Evaluation
	Levels of influence targeted	Primary intervention outcome	Was there an intervention effect? ^**^	Assumed inter-level process chains that are amenable to intervention?	Are stakeholders’ views on the assumed process chain assessed?	Real world context?	Across the targeted levels of influence, what background factors were considered in evaluation of the intervention to enhance the generalizability of effectiveness findings?	Was sustainability discussed?
Ehrbar V, et al (2019) [25]	• Individual • Interpersonal	Decisional conflict	Yes	The availability of counseling and online decision support materials will increase patient knowledge about fertility preservation options and lessen the likelihood of decisional conflict and regret	Not noted	Yes – Fertility centers	Sociodemographic characteristics of the patient were considered	Yes – Online decision aid materials can easily be updated
Ehrbar V, et al (2021) [26]								
Kelvin JF, et al (2016) [28]	• Individual • Interpersonal • Organizational	Number of patients receiving a consultation with a fertility clinical nurse specialist Patient satisfaction with fertility-related information received and the amount of information received	Yes	Available fertility-related educational materials for patients will increase fertility discussions with clinicians The existence of a provider referral network, resources and education for clinicians, and financial assistance for patients will increase referrals and consultations with fertility clinic nurse specialists	The survey instrument was developed with items based on relevant literature and the multidisciplinary clinical expertise of the investigators. It was also pilot tested with 10 patients of each gender and refined based on patient feedback	Yes – Cancer center	Sociodemographic characteristics of the patient	Yes – Considerations of strategies for prompting clinicians to use resources and provide materials to their patients
Partridge AH, et al (2019) [29]	• Individual • Interpersonal	Discussion of fertility within 3 months of initial appointment indicated in medical record	No	Educational materials provided to patients and providers will increase knowledge of fertility preservation and cue providers to have and document discussions with women who want to consider fertility preservation and increase patients’ satisfaction with cancer care. These processes will increase providers’ attention to fertility	Not noted	Yes – Academic institution and community sites	Sociodemographic characteristics of the patient; Differences in attention to fertility at community vs. academic practices were considered	No
Peate M, et al (2012) [27]	• Individual	Decisional conflict	Yes	Decision support materials will increase patients’ ability to make an informed decision regarding fertility preservation and lessen the likelihood of decisional conflict and regret	Focus groups were conducted with young women aged <45 (Ali & Warner, 2013) The decision aid was pilot tested with the target audience and was further refined prior to use in the intervention (Peate 2011b)	Yes - Oncology clinics	Sociodemographic characteristics of the patient	Yes – Considerations to transition to an online decision aid for ease of updating and lowering costs
Bradford NK, et al (2018) [30]	• Individual • Interpersonal • Organizational/Practice	Documentation of fertility-related discussions and referral for fertility preservation	Yes	Targeted oncofertility education for providers and additional educational resource packets for patients will prompt fertility-related discussions and documentation of such discussions Formalization of the referral process will increase consistency in provider referrals	Provider education sessions were based on a learning needs survey that assessed fertility and genetics knowledge; communication; sexuality, intimacy; and fertility preservation methods	Yes – Tertiary cancer centers	Literacy level differences by regions	No
Srikanthan A, et al (2016) [31]	• Individual • Interpersonal • System	Documentation and patient report of fertility-related discussion	Yes	Nurse coordinated support for patients will increase the frequency and documentation of fertility-related discussions with patients	The program was created by an interdisciplinary steering committee including representation from medical, radiation, surgical oncology, nursing, psychology, social work, and young breast cancer survivors A large advisory board also provided expertise on related topics (Ali 2013)	Yes – Cancer center clinics	Sociodemographic characteristics of the patient	No, however exploring a sustainable alternative to a nurse navigator
Vu J, et al (2017) [24]	• Individual • Interpersonal • Organizational	Discussions about treatment-related infertility and fertility preservation options	Yes	Improved patient and provider knowledge and EMR support and patient navigator will increase fertility-related discussions	Not noted	Yes – Cancer center	Sociodemographic characteristics of the patient and patient demographic makeup of the study cancer center over time	No, however exploring adding a decision-tree based prompt to the EMR to facilitate provider-led discussions
Balcerek M, et al (2020) [33]	• Individual • Interpersonal	Adolescent and parent fertility knowledge Adolescent and parent empowerment to make decisions regarding fertility preservation	Yes – Only for patient and parent empowerment	Informational cues to patients and their parents to become aware of fertility impairment risk will prompt discussions with providers and increase patient and parent knowledge and empowerment to engage in thoughtful decision- making for fertility preservation	Not noted	Yes - Pediatric oncology departments and clinics	None mentioned	No
Borgmann-Staudt A, et al (2019) [32]								
Warner E, et al (2020) [34]	• Individual • Interpersonal • Regional	Frequency of fertility-related discussion	Yes	Increased provider knowledge of oncofertility will improve surgeons’ abilities to have fertility-related discussions	A pre-intervention assessment with breast surgeons on oncofertility knowledge, attitudes, and practices was conducted to inform the resources included in the toolbox	Yes – Healthcare practices	Sociodemographic characteristics of the patient and practice characteristics	No

*Articles are shown in order of quality assessment

^**^Significant intervention effects for the primary outcome were considered

## Discussion

We reviewed fertility preservation intervention studies targeting reproductive-aged women to assess their alignment with ASCO practice guidelines; and whether they considered multilevel influences on service provision, effectiveness, and sustainability for healthcare system integration. Previous studies have shown that not adhering to evidence-based guidelines leads to practice variation and subsequently to a suboptimal quality of care and quality of life in survivors [34].

We found very few fertility preservation interventions included comparison groups which limits the rigor of the current evidence base. This review additionally identified gaps in the provision of ASCO guideline-concordant care. Our review suggests that while most interventions targeted some elements of the ASCO guideline, overall guideline adherence was limited and variable. Moreover, some interventions employed guideline relevant strategies but did not assess related outcomes. In these cases, it was unclear whether the interventions were effective in promoting guideline adherence.

The variability in alignment with ASCO guidelines may be associated with health care provider specialty and practice setting. Cancer care is provided by an interdisciplinary team of providers who see patients at varying points in the diagnosis and treatment process. Mapping fertility preservation intervention strategies to the standard course of care and the interchange between different care providers should be considered and tested in future interventions.

Additionally, greater attention should be given to specifically linking intervention strategies and evaluation outcomes to clinical practice and national guidelines. For example, Anazodo et al (2019) suggested the following: that the role and scope of practice for fertility care be defined for all healthcare providers, communication paths between different healthcare providers be mapped, discussions be initiated in a specified time frame, patients receive high quality communication to convey fertility risk and preservation options in different formats and at appropriate literacy levels; and that all communications and referrals to supportive care be documented [35]. Interventions that target these steps and measure related outcomes are needed to establish evidence-based care and standardized provision of fertility preservation services.

Provision of fertility preservation services also must be contextualized as it relies on individual (e.g., patient decision-making), interpersonal (e.g., family involvement and health care provider), organizational systems (e.g., staffing, electronic medical records), and societal (e.g., structural barriers to access). Thus, fertility preservation interventions must consider multilevel frameworks in developing strategies and evaluating outcomes. While we found that most interventions targeted at least two levels of influence, all analyses were conducted at the patient-level. Future intervention trials should be designed to report hierarchical outcomes. For example, applying innovative implementation science frameworks for intervention development, analysis, and evaluation efforts would be useful in this endeavor.

In considering the multilevel complexity of fertility preservation, it was surprising that very few interventions reported engaging stakeholders at any level of influence in guiding intervention development. Among the few that did, some engaged patient focus groups and structured interviews, others targeted health care providers. Further, stakeholder input was often considered prior to intervention implementation not during and after. Without continued stakeholder engagement to assess intervention progress and whether modifications need to be made, this could limit sustainability.

To develop interventions that are relevant, feasible, and sustainable, a more integrated and multi-level stakeholder engagement effort will be needed to identify linkages between levels of influence and mechanisms that might be amenable to intervention [23]. For example, a recent study found national guidelines often do not advise on the skills required for adherence. Building oncofertility competencies as outlined in the International Oncofertility Competency Framework will rely on integrating patient and family perspectives, provider resources, and organizational supports [7]. Thus, stakeholders at all levels have perspectives that will be important for an intervention to be sustainable.

There are some limitations to this review that warrant attention. First, we based our guideline alignment on ASCO clinical practice guidelines. It is notable that there are not yet a single set of unified guidelines for oncologists and fertility specialists for addressing fertility preservation [36]. Several other national and international organizations (e.g., ASRM, NCCN) have developed recommendations and guidelines to support cancer patients in preserving their fertility. There are common elements across these guidelines (e.g., timeliness of provider discussion, informed decision-making) that we felt were consistent with the ASCO guidelines. Moreover, we also focused on the broad categories that arise in the clinical process of care for fertility preservation and exclude those more distal to care such as provider encouragement of patients to participate in clinical registries. By narrowing the scope, we may have missed some of nuances of adherence to guidelines aimed to support the research process. Finally, there is additionally a possibility that our key words did not catch all intervention studies available in the literature.

## Conclusion

By 2023, the WHO estimates that 1.4 million reproductive-aged women will be diagnosed with cancer annually. As a result of scientific advances, increases in cancer survival rates have also led to the rising recognition for the field of Oncofertility and the need for fertility preservation. As seen in the literature, fertility preservation is still relatively new and evolving clinical area at the intersection of the fields of oncology and reproductive endocrinology. Yet, we can anticipate based on other new treatment implementation that systematic processes for delivering these services equitably will be needed [36]. This review identified key gaps in ASCO guideline-concordant care provision and the emphasized the importance of utilizing multilevel implementation frameworks for effective and sustainable interventions. The results of this review will allow future research to improve access, advancing research, educating oncofertility service providers, and collaborating with transdisciplinary members of the field to develop standardized processes of care [37, 38].

### Supplementary information

ESM 1 (DOCX 43 kb)
