# Supplementary material for: Evaluating fertility preservation interventions for alignment with ASCO Guidelines for reproductive aged women undergoing cancer treatment: a systematic review
Source: Support Care Cancer. 2023 Nov 11;31(12):689. doi: 10.1007/s00520-023-08133-3 (PMC10638151; doi:10.1007/s00520-023-08133-3)
Supplement: Supplementary file 1 — (DOCX 43 kb) [file 520_2023_8133_MOESM1_ESM.docx]

**APPENDIX 1. Detailed search criteria**

| **Database** | **Search Terms** |
| --- | --- |
| **Pubmed:** Filtered for female, human studies, English language, and publication date (2007-2022) | (((Neoplasm* OR oncology OR cancer) AND (Fertility preservation OR oncofertility OR "Fertility preservation")) AND (Health service* OR health care utilization OR health services research OR health care delivery OR health policy)) AND (Intervention) |
| **Embase:** No filters applied | (neoplasm*:ti,ab,kw OR oncology:ti,ab,kw OR cancer:ti,ab,kw) AND ('fertility preservation':ti,ab,kw OR oncofertility:ti,ab,kw) AND ('health care utilization':ti,ab,kw OR 'health services research':ti,ab,kw OR 'health care delivery':ti,ab,kw OR 'health policy':ti,ab,kw) AND intervention:ti,ab,kw |
| **Web of Science:** Refined by publication date (2007 to 2022) and English language | Neoplasm* OR oncology OR cancer (All Fields) and Fertility preservation OR oncofertility OR "Fertility preservation” (All Fields) and Health service* OR health care utilization OR health services research OR health care delivery OR health policy (All Fields) and Intervention (All Fields) |
| **Scopus:** Filtered for English language | ( TITLE-ABS-KEY ( neoplasm* OR oncology OR cancer ) AND TITLE-ABS-KEY ( oncofertility OR "fertility preservation" ) AND TITLE-ABS-KEY ( "Health service" * OR "health care utilization" OR "health services research" OR "health care delivery" OR "health policy" ) AND TITLE-ABS-KEY ( intervention ) ) |
